# Supercoiled DNA and non-equilibrium formation of protein complexes: A quantitative model of the nucleoprotein ParB*S* partition complex

**DOI:** 10.1371/journal.pcbi.1008869

**Published:** 2021-04-16

**Authors:** Jean-Charles Walter, Thibaut Lepage, Jérôme Dorignac, Frédéric Geniet, Andrea Parmeggiani, John Palmeri, Jean-Yves Bouet, Ivan Junier

**Affiliations:** 1 Laboratoire Charles Coulomb (L2C), Univ. Montpellier, CNRS, Montpellier, France; 2 CNRS, Univ. Grenoble Alpes, TIMC, Grenoble, France; 3 LPHI, Univ. Montpellier, CNRS, Montpellier, France; 4 LMGM, CBI, CNRS, Univ. Toulouse, UPS, Toulouse, France; University of Maryland School of Pharmacy, UNITED STATES

## Abstract

ParAB*S*, the most widespread bacterial DNA segregation system, is composed of a centromeric sequence, *parS*, and two proteins, the ParA ATPase and the ParB DNA binding proteins. Hundreds of ParB proteins assemble dynamically to form nucleoprotein *parS*-anchored complexes that serve as substrates for ParA molecules to catalyze positioning and segregation events. The exact nature of this ParB*S* complex has remained elusive, what we address here by revisiting the Stochastic Binding model (SBM) introduced to explain the non-specific binding profile of ParB in the vicinity of *parS*. In the SBM, DNA loops stochastically bring loci inside a sharp cluster of ParB. However, previous SBM versions did not include the negative supercoiling of bacterial DNA, leading to use unphysically small DNA persistences to explain the ParB binding profiles. In addition, recent super-resolution microscopy experiments have revealed a ParB cluster that is significantly smaller than previous estimations and suggest that it results from a liquid-liquid like phase separation. Here, by simulating the folding of long (≥ 30 kb) supercoiled DNA molecules calibrated with realistic DNA parameters and by considering different possibilities for the physics of the ParB cluster assembly, we show that the SBM can quantitatively explain the ChIP-seq ParB binding profiles without any fitting parameter, aside from the supercoiling density of DNA, which, remarkably, is in accord with independent measurements. We also predict that ParB assembly results from a non-equilibrium, stationary balance between an influx of produced proteins and an outflux of excess proteins, i.e., ParB clusters behave like liquid-like protein condensates with unconventional “leaky” boundaries.

## Author summary

In bacteria, faithful genome inheritance requires the two replicated DNA molecules to be segregated at the opposite halves of the cell. ParAB*S*, the most widespread bacterial DNA segregation system, is composed of a centromere sequence, *parS*, and two proteins, the ParA ATPase and the ParB DNA binding protein. Hundreds of ParB assemble dynamically to form clusters around *parS*, which then serve as substrates for ParA molecules to catalyze the positioning and segregation events. The nature of these clusters and their interaction with DNA have remained elusive. Here, we propose a realistic minimal model that captures quantitatively the peculiar DNA binding profile of ParB in the vicinity of *parS* in *Escherichia coli*. From the viewpoint of DNA, the only fitting parameter is the *in vivo* supercoiling density resulting from the removal of DNA helices by toposiomerases, which is in accord with previous independent estimations. From the viewpoint of ParB clusters, we predict that they behave like liquid-like protein condensates with unconventional boundaries. Namely, we predict boundaries to be leaky (i.e. not sharp) as a result of the non-equilibrium protein production, diffusion and dilution. Altogether, our work provides novel insights into bacterial DNA organization and intracellular liquid-liquid phase separation.

## 1 Introduction

Bacteria display many mechanisms to control and position precisely and specifically macromolecular complexes in their cellular environment. Some of these mechanisms use the nucleoid as a matrix [1, 2] like, e.g., the system PomXYZ (cell division site positioning [3]) or McdAB (carboxysomes positioning [4]). Here, we investigate the case of the ParAB*S* system essential for the stable inheritance of most chromosomes and low-copy-number plasmids [5, 6]. Specifically, ParAB*S* uses a force dipole of chemical origin that acts on replicated DNA molecules to separate them from each other [7–12], ensuring the faithful genomic inheritance between daughter cells. It is composed of a centromeric sequence, *parS*, and two proteins, the ParA ATPase and the ParB DNA binding proteins: hundreds of ParB proteins assemble dynamically to form nucleoprotein *parS*-anchored complexes (called clusters below) that serve as substrates for ParA molecules to catalyze positioning and segregation events [7–12]. More specifically, the nucleation process of these clusters starts with a few ParB proteins (typically one to ten) that bind to 16 bp long *parS* sites. This initiates the assembly of hundreds of ParB dimers in the close vicinity of *parS*, which are visible as intense foci in fluorescence microscopy [13].

On the one hand, in agreement with the observation of a network of low but synergistic ParB-ParB and ParB-DNA interactions [13], super-resolution microscopy experiments have revealed that the nucleation process involves a droplet-like assembly of the hundreds of ParB *via* liquid-liquid phase separation [14]. Yet, a precise description of the resulting clusters remains beyond the current capacity of microscopy experiments, i.e., the exact nature of these clusters remains open. On the other hand, chromatin immunoprecipitation sequencing (ChIP-seq) experiments have revealed the existence of a specific DNA binding profile around *parS* [13, 15]. We have previously shown that this profile can be explained by invoking a process where DNA looping stochastically brings loci inside a cluster with sharp edges [13, 15], corresponding, in this article, to a quenched cluster (see below). This so-called stochastic binding model (SBM) has nevertheless been calibrated using a worm-like chain description of DNA devoid of the torsional constraints typical of *in vivo* conditions. As a consequence, in order to match experimental data, the SBM required microscopic parameters difficult to justify on physical grounds, namely, a very small DNA persistence length of ∼ 10 bp [13, 15]. In addition, modeling has been made with the width of the ParB cluster equal to 150 nm (previous limit of microscopy resolution), whereas the most recent super-resolution microscopy experiments leads to an estimation on the order of 40 nm [14]—the use of the latter estimate would decrease even further the persistence length needed to model the data. Finally, at short genomic distance from *parS*, the drop observed in the ChIP-seq profile between the specific binding *parS* region and the non-specific binding DNA region was modeled considering different treatment of the ChIP-seq signal for these regions. However, super-resolution microscopy experiments revealed a ParB concentration in the cluster of the order of 20 mM [14], which is much larger than the dissociation constant of ParB with non-specific DNA (0.5 *μ*M) and, hence, requires to treat specific and non-specific binding sites similarly.

**Fig 1:**
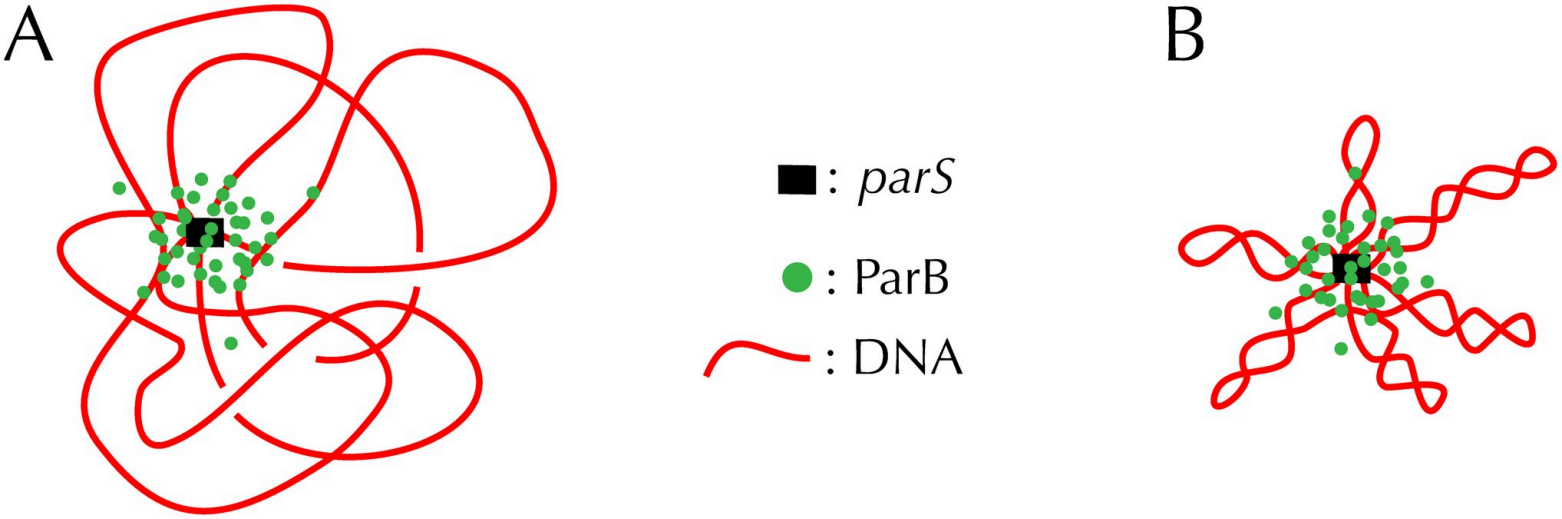
Stochastic binding model. When DNA enters the high concentration region of the *parS*-anchored cluster of ParB, crosslinking with ParB occurs with high probability during the ChIP-seq protocol. Compared to relaxed DNA (A), supercoiling (B) tends to increase DNA compaction and, hence, crosslinking with DNA loci far from *parS*. See Fig D in S1 Text for conformations resulting from our simulations.

In this article, we show that an out-of-equilibrium formation of the ParB cluster together with a realistic model of bacterial DNA including its torsional properties leads the SBM to correctly capture the different measurements associated with both ChIP-seq and super-resolution microscopy data. Regarding bacterial DNA, we recall here that, *in vivo*, it is continuously processed by topoisomerases [16], whose activity allows relaxing the transient constraints generated by DNA replication and by gene transcription. As a result, bacterial DNA is generally underwound, a property that plays a critical role for genome structuring and coordination of gene expression [17–20]. As sketched in Fig 1, this so-called negative supercoiling of DNA strongly influences long range DNA spatial organisation as a result of topological constraints imposed by the DNA double-helix. Namely, a circular DNA molecule such as a plasmid is characterized by a constant linking number, Lk = Tw + Wr, where the twist Tw is the cumulative helicity of the molecule, and the writhe Wr is the number of loops made by the axis of the molecule around itself [21]. At rest, the linking number, Lk_0_, is equal to the average twist (the number of B-DNA helices along the contour length) and Wr ≃ 0. Supercoiling, as a result of the action of topoisomerases, corresponds to Lk ≠ Lk_0_ and is commonly characterized by the supercoiling density, *σ* = (Lk − Lk_0_)/Lk_0_, the relative variation of Lk with respect to Lk_0_. Specifically, whenever *σ* ≠ 0 (supercoiled DNA), there exists an equilibrium between non-zero values of Wr associated with compact plectonemic super-structures (Fig 1, see also Fig D in S1 Text) and twist values different on average from Lk_0_ [22]. In the case of bacteria living in mild environments, *σ* < 0 (Lk < Lk_0_) meaning that the helix is underwound—typical values in *Escherichia coli* are on the order of −0.06 [23].

For the previous SBM to match experimental data, the requirements of both a small persistence DNA length and a large nucleoprotein complex reflected a too swollen DNA conformation around *parS*. Here, using numerical simulations of realistic long (i.e. ≥ 30 kb) molecules, we show that the natural compactification induced by *in vivo* relevant DNA supercoiling properties in the context of an out-of-equilibrium formation of the ParB cluster solves these problems. Moreover, DNA supercoiling provides a rationale for the drop observed in the ChIP-seq profile at short genomic distance from *parS* and, hence, does not require, as in the previous SBM, to consider different treatment of the ChIP-seq signal depending on whether binding to DNA is specific or not. Namely, DNA supercoiling induces the formation of plectonemes that are more rigid than the DNA molecule (typically twice the persistence length) so that DNA actually exits the cluster quickly at small genomic distances. Finally, we predict a cluster shape that differs from the usual sharp boundaries of liquid droplets. Namely, to capture quantitatively the experimental results, one must use a cluster density profile that displays unconventional “leaky” boundaries, which can be explained as a perturbation induced by a source of proteins located at the edge of the cluster core. Using our modeling framework, we also provide a bound for the chromosomal supercoiling density *in vivo* in accord with classical measurements on a plasmid. Altogether, our work thus both provides insights into liquid-like protein condensates and opens the way to quantitatively measuring local chromosomal supercoiling.

The paper is organised as follows: in section 2, we present the general formulation of the SBM. In section 3, we present the polymer model of supercoiled DNA and explain the simulation procedure. Section 4, the main results part, is divided into three subsections. In subsection 4.1 we confront our model predictions with experiments, focusing more particularly on two extreme situations for the ParB cluster: i) a quenched cluster with sharp borders and ii) a leaky cluster resulting from an out-of-equilibrium process of spatially localized production, diffusion and dilution of proteins. In 4.2, we provide additional evidence for the relevancy of the leaky cluster based on the prediction of the total number of ParB inside the cell. In 4.3, we discuss properties of the supercoiled conformations that allow to match experimental data. We conclude with a discussion of the results in section 5.

## 2 Stochastic binding model

In the SBM, the non-specific binding of ParB around *parS*, as detected using ChIP-seq experiments, results from looping properties of DNA that bring genomic loci into the ParB cluster [13, 15]. This binding profile around *parS* is simply expressed in the SBM as the integral over space of the product between the concentration of ParB proteins at a given point and the probability to find loci at this point (Eq 1) [13, 15]. To understand this relationship, let us first recall that ChIP-seq detection of DNA-bound proteins involves sub-nm crosslinking between DNA and proteins [24]. As a consequence, one can expect that the non-specific ParB binding profile results from “collisions” between DNA and the ParB proteins located in the *parS*-anchored cluster (Fig 1). Then, supposing that the timescale for ParB to unbind DNA is much shorter than the timescale for DNA to diffuse away from the location where binding occurs (instantaneous unbinding hypothesis), the modeled non-specific ParB binding profile, *B*(*s*), reads:

$$\begin{array}{c} B\left(s\right)=\int 4\pi r^{2}P_{s}\left(r\right)C\left(r\right)dr\;. \end{array}$$ (1)

*P*_*s*_(*r*) describes DNA “looping properties”: it stands for the equilibrium probability distribution function for a DNA locus at a genomic distance *s* from *parS* to be located at a distance *r* from *parS* in the three-dimensional space. For simplicity, here we neglect effects coming from the interaction between DNA and the cluster, therefore *P*_*s*_(*r*) is computed by considering an isolated DNA chain. This approximation is all the better that DNA loci are located away from *parS* (see below for further discussion). Note, finally, that the divergence of the integral in Eq 1 is prevented by a fast decay of *P*_*s*_(*r*, *s*) for large *r* and the finite volume of integration (see section 4.2 for further details).

*C*(*r*) stands for the probability, during the time window associated with the crosslinking stage of the ChIP-seq measurement, for a point located at distance *r* from *parS* to be in the presence of a ParB protein. Although its exact shape is not known (see below for predictions), we have *C*(*r* = 0) = 1 by definition of the strong binding of ParB to *parS*. Next, the full width at half maximum of the cluster, *ω*, has been estimated using super-resolution fluorescent microscopy, leading to ω_*exp*_ = (37 ± 5) nm [14]. In these super-resolution experiments, Brownian motion is suppressed by using a fixating agent that freezes the content of the cell. Clusters identified in multiple measurements are therefore not necessarily centered on *parS* since there is motion of DNA inside the cluster [14]. A proper treatment of the problem thus requires, *a priori*, to distinguish the cluster that is measured in these experiments from the effective *parS*-centered cluster that is associated with *C*(*r*), the latter including Brownian motion of the former. Specifically, *ω*_*exp*_ refers to *C*^(0)^(*x*), the probability during the crosslinking stage for a point at distance *x* from the cluster center (i.e. not from *parS* *a priori*) to be in the presence of a ParB protein—in particular, $C_{exp}^{\left(0\right)}\left(\omega_{exp}/2\right)=0.5$. Considering the positional degrees of freedom of the cluster center with respect to *parS*, we can nevertheless write $C\left(r\right)=\int_{0}^{\infty }dx\;\Pi_{r}\left(x\right)C^{\left(0\right)}\left(x\right)$ for the effective cluster, where Π_*r*_(*x*) stands for the probability density of finding the center of the cluster at a distance *x* given a point at distance *r* from *parS* (Material and methods). We note, nevertheless, that considering *C*^(0)^(*r*) as an approximation for *C*(*r*) leads to similar results, with significant differences only for small binding probabilities.

## 3 Self-avoiding rod-like chain model of DNA

In contrast to the previous version of the SBM [13, 15], we consider a realistic 30 bp resolution polymer model of bacterial DNA, namely the self-avoiding rod-like chain (sRLC) model [22] (see detailed simulation procedure in [25]). Specifically, DNA is modeled as a discrete chain of 10.2 nm long (30 bp of B-DNA) hard-core cylinders, with radius *r*_*e*_ = 2 nm reflecting the short-range electrostatic repulsion of DNA for *in vivo* salt conditions [26]. Contiguous cylinders can rotate around their common extremity, hereafter called an articulating site, and the chain is iteratively deformed using crankshaft elementary motions with Metropolis-Hastings transition rates, under the condition that it does not cross itself. Each articulating site is associated with bending and torsional energies such that the resulting persistence length (50 nm or, equivalently, 147 bp) and torsional stiffness (86 nm) are typical of B-DNA for *in vivo* salt conditions [22, 27]. In accord with recent single molecule measurements (see e.g. [28]), we note that an intrinsic torsional stiffness of 86 nm leads to an effective torsional stiffness (the value a straight rod should have to yield the same torque) between 60 and 80 nm when DNA is stretched by forces below 1 pN [26]—here, we do not consider stretching.

In this article, we discuss results obtained with a 30 kb long chain simulated using an annealing procedure (Material and methods). In short, numerical simulations consist in starting from an initial circular conformation of DNA with *σ* = 0 (Lk = Lk_0_ = 2850 helices) and in decreasing *σ* down to *σ* = −0.08 in a stepwise manner by removing 14 helices, which corresponds to a decrement Δ*σ* ≃ −0.005. After each removal of helices, the chain relaxes at constant *σ* as a natural consequence of chain circularity and self-avoidance [25]. We then perform an analysis by increasing the window time during which *σ* is kept constant, which is equivalent to decreasing the rate of change of *σ*. Using this simulated annealing procedure, we present results obtained with a rate of change of *σ* that is small enough so that chain statistical properties are insensitive to it (See section 6.1 in Material and methods). These simulated conformations are thus expected to reflect thermodynamic equilibrium, even at low values of *σ* where plectonemes are tight. We further checked that our results did not depend significantly on the length of the chain by performing additional simulations of 60 kb long chains (Fig A in S1 Text). Note, here, that the motivation to work with *σ* ≥ −0.08 is both biological and physical: in the worst case of topoisomerase mutants, the total supercoiling density in *E. coli* has been shown to remain above −0.08 [23], while recent work has revealed the existence of a transition toward a hyperbranched regime occurring at *σ* ≃ −0.08 [29], which is beyond the scope of our discussion.

## 4 Results

### 4.1 Leaky vs quenched cluster

Having in hand the corresponding *P*_*s*_(*r*) for *σ* ∈ [−0.08, 0], we now consider the spatial distribution of ParB proteins associated with the *parS*-anchored clusters. Super-resolution microscopic measurements suggest that these clusters result from a phase transition-like mechanism [14]. Theoretical models further suggest that this phase transition is unconventional as it may imply a framework of a lattice gas on a fluctuating polymer [30]. From a biological perspective, the mechanisms underlying the formation of a cluster should reflect the non-trivial cellular organization of genetic information and proteins [31]. Altogether, this means that the exact spatial distribution of ParB proteins around *parS* remains an open problem. Yet, in *E. coli*, the production of proteins at the place where they are required is a frequent phenomenon [32, 33]. As a consequence, it is reasonable to hypothesize that ParB proteins are synthesized close to the cluster—see below for a thorough analysis.

**Fig 2:**
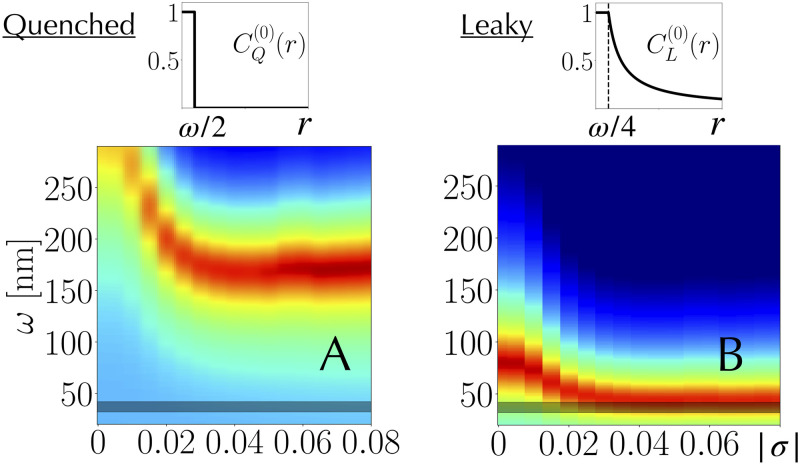
Capturing chromosomal binding profiles. Root mean squared deviation between modeled binding profiles and ChIP-seq chromosomal data (curves can be found in Fig B in S1 Text); the redder the pixel, the smaller the deviation (arbitrary scale). The horizontal dark band indicates *ω*_*exp*_(37 nm ± 5 mn). (A) The best models with quenched clusters imply a large cluster with *ω*_*best*_ = 170 nm. (B) In contrast, the best models with leaky clusters imply cluster sizes very close to those extracted from microscopic data when *σ* ≲ −0.04. In this regime, all best models indeed correspond to *ω*_*best*_ = 44 nm.

Here, we investigate more particularly two extreme cases for the shape of these clusters, referred to as *quenched* and *leaky*. A quenched cluster (Fig 2A) is defined by $C_{Q}^{\left(0\right)}\left(r\right)=\theta \left(\omega /2-r\right)$, with *θ* the Heaviside function. It corresponds to the conventional sharp interface of a droplet. A leaky cluster (Fig 2B) further includes the stationary solution of a diffusion process where ParB proteins are continuously produced at the edge of the cluster core and diluted due to cell growth and division (Material and methods). That is, the leaky cluster releases proteins in excess, while $C_{L}^{\left(0\right)}=1$ for $r\leq \frac{\omega }{4}$ (cluster core) reflects the saturation regime in which experiments are performed [15]. As a solution of the stationary diffusion/dilution equation, we find that $C_{L}^{\left(0\right)}$ is well approximated by a 1/*r* long range decay inside the cell such that $C_{L}^{\left(0\right)}\left(r\right)=\theta \left(\frac{\omega }{4}-r\right)+\frac{\omega }{4r}\theta \left(r-\frac{\omega }{4}\right)$ (Material and methods). Finally, we investigated two additional intermediate situations where *C*^(0)^(*r*) decays according either to a Gaussian form, as in the previous SBM [13, 15], or to an exponential form. Note that for all cases, the full width at half maximum of *C*^(0)^ is equal to *ω*.

We computed ParB DNA binding profiles for *σ* ranging in [−0.08, 0] and for values of *ω* between 10 nm and 300 nm. We compared them with DNA binding profiles of ParB_F_ (referred to as ParB in the following for simplicity), which specifically binds the centromere of the F plasmid (*parS*_F_). To this end, we used previously published ChIP-seq data of the binding of ParB in the vicinity of the *parS*_F_ locus in *E. coli* [13, 15]. Specifically, the *parS*_F_ locus is composed of 10 specific ParB binding sites interspersed by 43 base pairs [34]. Importantly, the corresponding ParB DNA binding profiles were highly reproducible. Interestingly, they only weakly depend on the DNA molecule onto which *parS*_F_ is located, that is, either at its natural location on the 100 kb long F plasmid (hereafter referred to as plasmid data) or at the *xylE* insertion locus on the *E. coli* chromosome (chromosome data). Moreover, while the level of the profile depends on the intracellular level of ParB, the form of the decay also depends only weakly on it [15].

For the present study, we introduced two noticeable modifications for the representation and normalization of the ChIP-seq data: (i) the reads were counted at the center of each fragment (knowing the average DNA fragment size for each library) instead of at their 5’ ends, and (ii) the normalization was performed with respect to the maximal value (genomic position varies slightly depending on the profiles) in contrast to previous normalizations either relative to the read value at a given position (the first nucleotide after the last ParB binding repeat [15]) or to the average number of reads over the whole *parS*_F_ region [13]. Lastly, as in previous studies, only one side of the DNA binding profile was analyzed as the other side is distorted by the presence of strong promoter regions for chromosome data, and roadblock proteins for plasmid data [13, 15]. The origin of the curvilinear abscissa *s* was set right at the edge of the most extreme *parS*_F_ site.

To quantify the explanatory power of each model, we report the root mean square deviation with respect to the experimental binding profile for *s* ∈ [1.5 kb, 9 kb]. The lower and upper bounds at 1.5 kb and 9 kb, respectively, are used to avoid specific, reproducible distortions of the signal associated with the presence of gene promoters and sites for regulatory DNA proteins [15].

We find that the four types of clusters (quenched, Gaussian, exponential and leaky) can accurately capture the experimental data on the *E. coli* chromosome (Fig B and C in S1 Text). However, the best quenched models are found at a much larger value than *ω*_*exp*_, on the order of the radius of gyration (Fig A in S1 Text): *ω*_*best*_ = 170 nm (Fig 2A). The same is true for the Gaussian and exponential cases, with *ω*_*best*_ = 125 nm and *ω*_*best*_ = 80 nm, respectively.

In contrast, the best leaky models are found at *ω*_*best*_ = 44 nm when *σ* ≲ -0.04 (Fig 2B). That is, they predict the right width of the cluster and explain data in the physiologically relevant plectonemic regime of bacterial DNA. By doing so, they provide a rationale for the necessity, in the previous version of the SBM [13], of having to use both a small DNA persistence length and a large width of the Gaussian cluster: the former was required to “mimic” the supercoiling induced DNA compaction (Fig D in S1 Text); the latter “solved” the too quick Gaussian decay.

**Fig 3:**
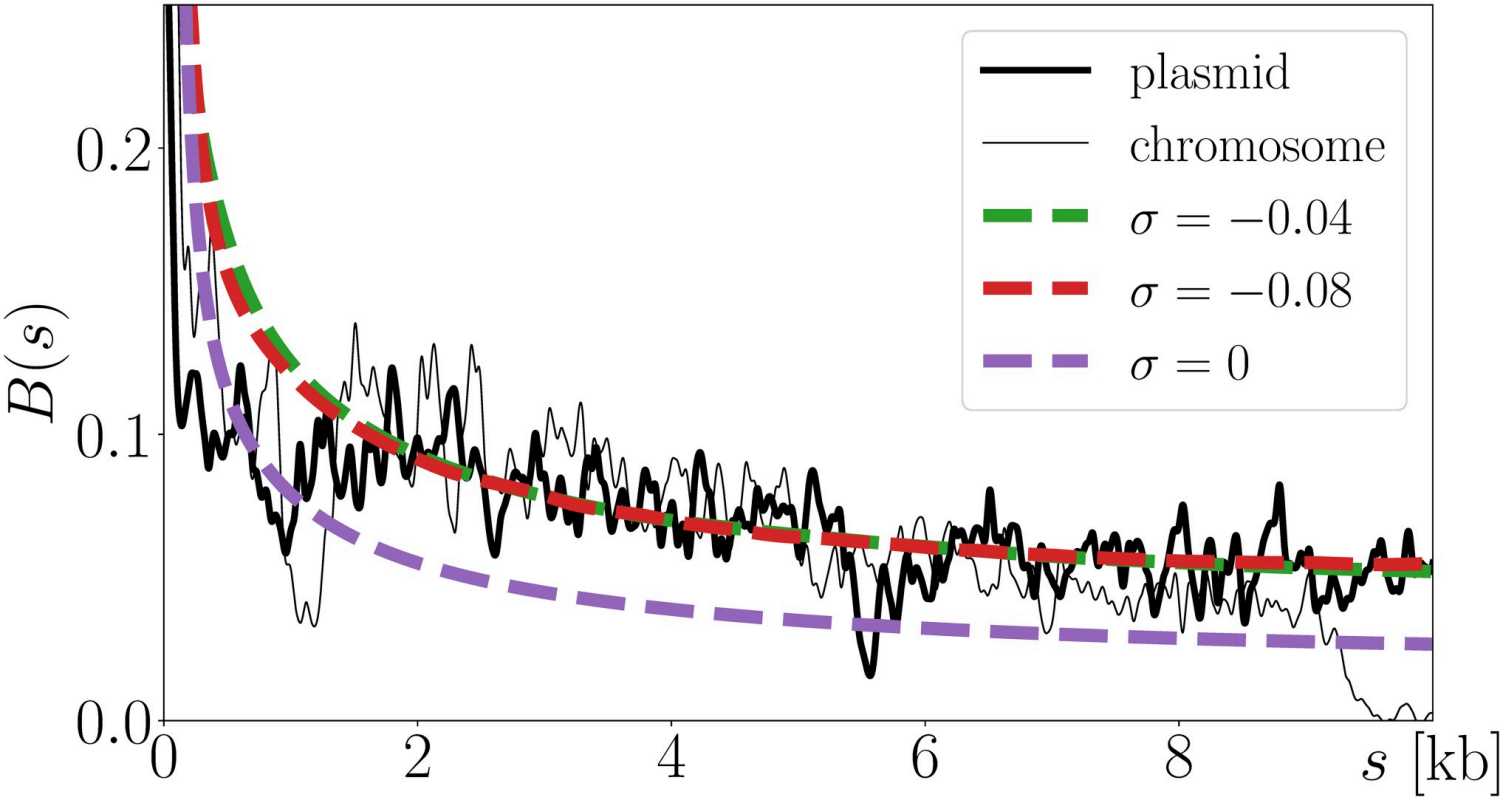
Non-specific ParB binding profile, B(s) (Eq 1). Compared to chromosome data (thin black curve), the leaky models with *ω*_*best*_ = 43 nm (smooth curves) and *σ* ≤ −0.04 (green and red curves) capture even better plasmid data (thick black curve). Predictions for *σ* = −0.04 and *σ* = −0.08 are almost indistinguishable, in accord with results of Fig 2.

Interestingly, compared to chromosome data, ParB DNA binding profiles in the vicinity of a *parS*_F_ located on the plasmid F show less distortion (Fig 3). In this context, the best leaky models lead to similar model parameters (*ω*_*best*_ = 43 nm when *σ* ≲ −0.04), while providing an even better match with the data (Fig 3). Compared to the chromosomal situation where the gene *parB* is located 750 kb away from *parS*, this better match might reflect a phenomenology of the plasmid fitting particularly well the leaky situation, with *parB* located only 74 bp away from *parS* [35]. The hypothesis of a source located on the edge of the cluster core is indeed even more relevant since the production of proteins in bacteria often occurs close to their genes [36, 37].

### 4.2 The leaky cluster model is consistent with the number of ParB proteins

To further check the consistency of the leaky cluster model with respect to the situation *in vivo*, one can compute the average number of proteins resulting from this model and compare it to experimental estimations. To this end, let first recall that *C*^(0)^(*r*) is the probability to find, during the time window associated with the crosslinking stage of the ChIP-seq measurement, a point in space located at a distant *r* away from the cluster center to overlap with a ParB protein (*C*^(0)^(*r*) = 1 means a protein is always found at that point). Given this definition, the average number of proteins, *N*_*P*_, in a spheric cell of radius *R* is given by $N_{P}=\int_{0}^{R}C^{\left(0\right)}\left(r\right)4\pi r^{2}dr/v$ where *v* is the effective volume of the protein in the context of the ChIP-seq process. For simplicity, we can consider a protein as a sphere of radius *a* such that *v* = 4*πa*^3^/3. Considering the explicit form of the leaky cluster ($C_{L}^{\left(0\right)}\left(r\right)$, see above), one finally obtains:

$$\begin{array}{c} N_{P}={\left(\frac{\omega }{4a}\right)}^{3}[1+\frac{3}{2}({\left(\frac{4R}{\omega }\right)}^{2}-1)] \end{array}$$ (2)

Supposing *R* = 400 nm (radius of the nucleoid), we respectively find *N*_*P*_ ≃ 17280, 2160, 640, 270 for *a* = 5, 10, 15, 20 nm. Knowing that there are of the order of a few hundreds of ParB proteins per complex in our experiment [13], this suggests *a* to be on the order of 15 − 20 nm. This size is therefore larger than the radius of a ParB protein (of the order of 5 nm). Yet, it remains in the molecular range. In this regard, we note that crosslinking protocols have been predicted to lead to the formation of pearl-like local structures [38] (even at low concentration of crosslinkers), which are expected to increase the cross section between DNA and proteins. We also note, here, that 20 nm is the typical distance accessible for a ParB in the complex [39, 40].

### 4.3 Properties of plectonemic super-structures

**Fig 4:**
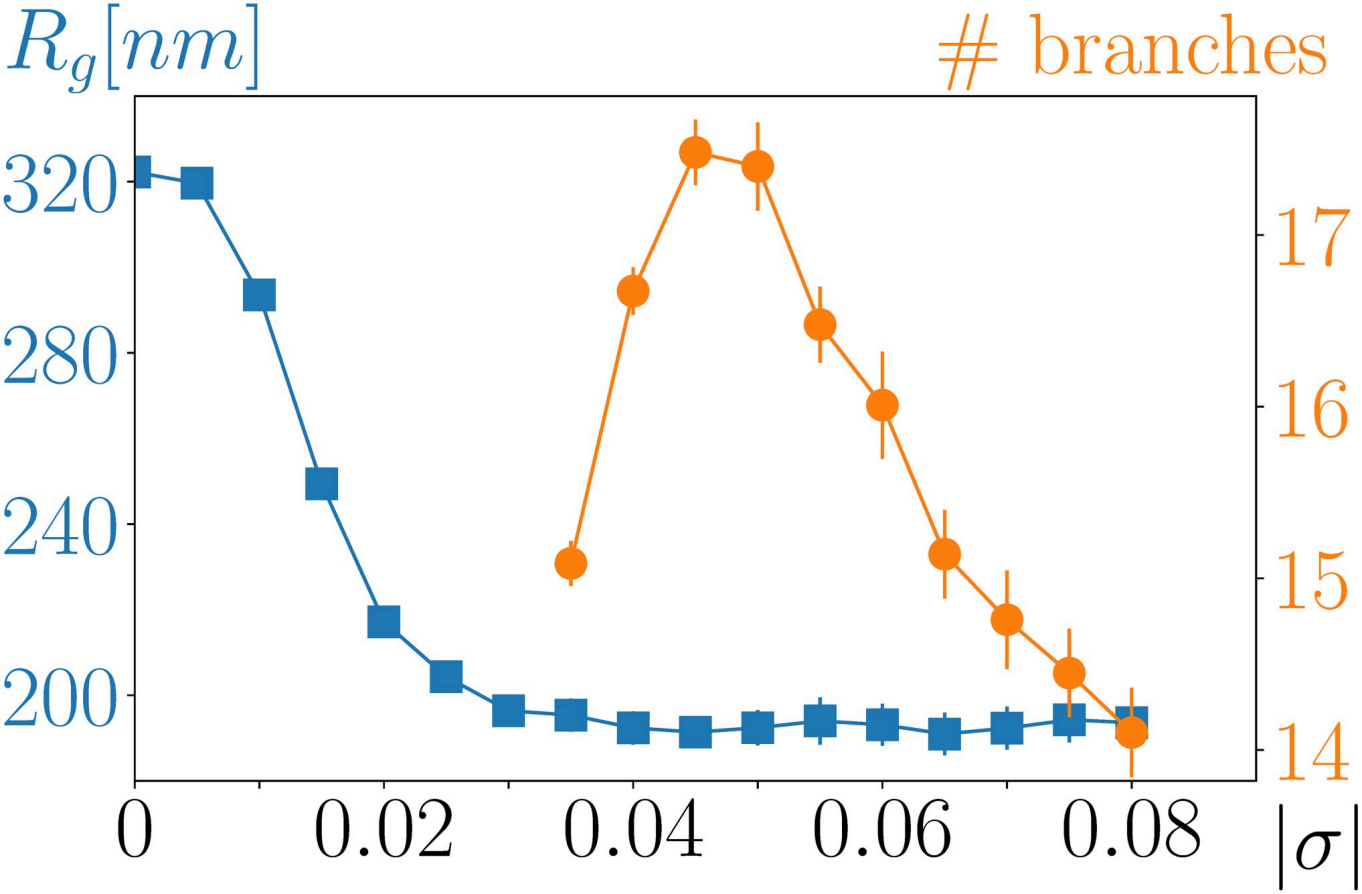
The radius of gyration of a 30 kb long circular molecule plateaus at |*σ*| ≃ 0.04. The number of plectonemic branches is non-monotonous, reaching a maximum at |*σ*| ≃ 0.05. The error bars correspond to the standard error of the mean computed over 20 different simulation runs (see Material and methods for details).

Interestingly, while leaky models with experimentally relevant *ω* capture the experimental data rather well, resulting binding profiles are almost indistinguishable for *σ* ∈ [−0.08, −0.04] (Fig 3). This observation is consistent with a weak variation of the radius of gyration (blue curve in Fig 4) in the plectonemic regime. In contrast, branching properties are expected to vary significantly in this regime [29, 41]. Here, we find that the number of plectonemic branches reaches a maximum at *σ* ≃ −0.05 (orange curve in Fig 4), in accord with previous analyses with smaller molecules [41] and with a minimum value of the hydrodynamic radius for 10 kb long plasmids [29, 41, 42]. Inspection of the snapshots of DNA conformation for various values of *σ* presented in Fig D in S1 Text suggests that the radius of gyration remains constant for |*σ*| ≥ 0.04 because the non-monotonous change in the number of plectonemic branches is compensated by variations in their average length.

## 5 Discussion and perspectives

Here, we have shown that the binding profile of ParB proteins in the vicinity of *parS*, i.e. below 10 kb, can be quantitatively explained considering an SBM involving supercoiled DNA and proteins that are issued from a saturated *parS*-anchored core cluster. To this end, we had to consider clusters from a non-equilibrium, stationary diffusion perspective, with the presence of a spatially localized source and a sink. Biologically, the sink reflects protein dilution due to cell growth and division, while the source may arise from two effects: the continuous activity of genes producing new proteins in a saturated cluster and the effect of an unconventional liquid-like nature of the cluster. Namely, we predict the cluster core to result from a balance between an influx of continuously produced proteins and an outflux of proteins in excess. This scenario calls naturally for the production of proteins to occur close to the cluster. This might happen in the chromosomal case (where *parS* is far from *parB*) as an effect of the spatial colocalization of the gene with the cluster, knowing that in bacteria protein production often occurs close to the place where they are required [32, 33, 43]. In plasmids, the situation may even be more prototypical since *parB* and *parS* are near each other along the DNA in this case and since transcription and translation are often tightly coupled in *E. coli* [33, 36, 37]. This would explain the better match between experiment and our model predictions in this case (Fig 4).

Next, to better understand the novelty of our approach with respect to the previous version of SBM, we recall that the SBM was developed to explain the slow decay of the ParB binding frequencies as the genomic distance *s* to *parS* increases. Namely, we previously showed that a spreading or a bridging mechanism could not explain this decay in the strong binding limit examined in detail up until now [13]. Instead, a mechanism where DNA motion stochastically brings loci inside the cluster was required to capture the specific shape of the decay. Yet, in order to explain the drop at small *s* (i.e. below ∼ 200 bp), our previous version of the SBM, which discarded supercoiling properties of bacterial DNA, included an *ad hoc* two-state mechanism that led, in effect, to a different amplitude of the ChIP-seq signal depending on the affinity of the binding of ParB on specific (at *parS*) or non-specific DNA [13]. In the light of recent experiments, the concentration of ParB inside the clusters is of the order of mM [14], which is much higher than the *K*_*d*_ of ParB for non-specific DNA, of the order of *μ*M. Thus, a two-state mechanism discriminating between specific and non-specific binding site is not justified. Moreover, to fit the exact level of the overall decay, we had to consider a persistence length of DNA (10 bp) much smaller than that expected *in vivo*, even by considering the action of nucleoid associated proteins that can bend and wrap DNA [44, 45]. Remarkably, our improved version of the SBM based on supercoiled DNA and a non-equilibrium formation of the ParB cluster solves both problems. Namely, on the one hand, supercoiling-induced plectonemes generate a natural drop as they allow DNA to exit quickly from the ParB cluster (large effective rigidity inside the plectoneme, see Fig D in S1 Text). On the other hand, our model leads to the right level of the binding frequencies without the need of a small persistence length, provided that the supercoiling density *σ* is below ≃ − 0.04.

Interestingly, this upper bound value for *σ*, which is found for the exponential growth of *E. coli* both along the chromosome and on the plasmid, corresponds to the onset of the plectonemic regime characterized by a weak variation of the radius of gyration, on one hand, and a significant variation of branching properties, on the other hand (Fig 4). It is then interesting to note that *in vivo* about 50% of the *E. coli* DNA supercoiling, which is known to be on the order of −0.06, is titrated by proteins [23, 46], i.e., by nucleoid associated proteins that absorb excess of writhe. Thus, the remaining so-called free supercoiling density *in vivo* is about −0.03. This value is close to the upper bound limit (−0.04) of the interval of supercoiling for which the leaky model shows the better match with the ParB DNA binding profile.

Interestingly, having a quantitative physical model of the local folding of bacterial DNA in interaction with ParB clusters opens the road to quantifying other protein binding properties that are directly related to biological processes and that compete with the binding of ParB, such as the binding of the RNA polymerase at promoters. For that matter, one would like to have an explicit description of ParB nucleation and diffusion properties in order to develop a detailed model of the interactions between ParB and DNA using e.g. molecular dynamics approaches. In particular, part of the discrepancy between experimental and modeling profiles below ∼ 1 kb (Fig 3) might be the result of our approximation of neglecting these interactions not only in computing DNA conformations, but also in estimating the resulting binding profile. Away from these short distances, the specific (still unknown) details of the interaction between DNA and ParB in the core of the cluster are expected to be irrelevant, justifying the relevance of our approach.

Finally, let us note that ParB was recently shown to belong to a new class of CTP-dependent (cytidine triphosphate) molecular switches [39]. Namely, ParB dimers bound to *parS* sites switch to a conformation that enables the binding to two CTP nucleotides. This subsequently induces the conversion of ParB into a clamp over the DNA and its subsequent release from *parS* [39, 40, 47]. Clamped-ParB may then slide over the DNA proximal to *parS* [39]. This clamping and sliding is expected to occur only over a limited distance and for a small number of ParB, as suggested by physical modeling [48]. Also, the presence of numerous barriers present over the DNA, such as protein-DNA complexes, would prevent clamped-ParB from sliding over the large genomic distance observed for both chromosome- and plasmid-encoded ParBs. Therefore, the ParB binding at large genomic distance from *parS* remains best explained phenomenologically by the SBM. In this regard, cellular confinement of DNA, especially for bacterial chromosomes, should be included in the model to fully account for the large genomic distance behavior. A complete picture would nevertheless require studying the melting of a plectonemic tree-like structure at the chromosomal scale, which is currently beyond the capacities of numerical simulations.

## 6 Material and methods

### 6.1 Simulation protocol

Thermodynamic properties of chains were investigated using a simulated annealing procedure. Namely, starting from a circular conformation at *σ* = 0, a simulation run consisted in repeating the following steps from *σ* = 0 down to *σ* = −0.08:

1. Perform N sweeps (cf. below for further discussion about this parameter).
2. Decrease *σ* by 0.005.
3. Goto 1.

Note here that due to the circularity of the chain and the fact that it cannot cross itself (self-avoidance), *σ* is constant in step 1 [25]. Step 2 is implemented by removing typically 14 helices (Δ*σ* ≃ −0.0049), which is done by rotating the associated Euler-like frame of cylinders such that the twist change at each articulating site is equal to −14/*N*, with *N* the number of cylinders (or sites, equivalently).

**Fig 5:**
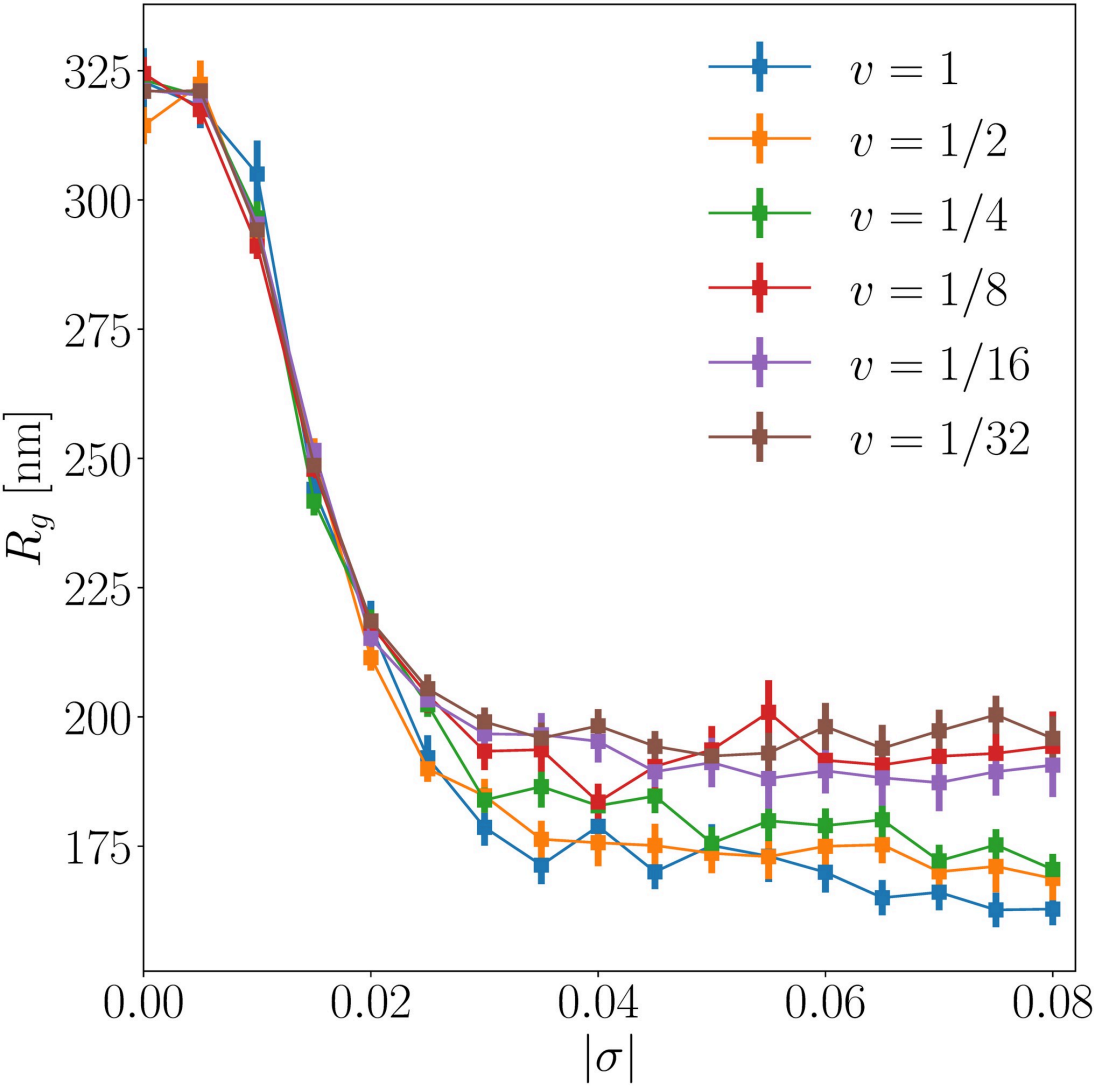
Sensitivity of results with respect to supercoiling variation rates. In this plot, a point corresponds to the mean value of the radius of gyration obtained at a given *σ* for a specific supercoiling rate *v* (see explanations for the protocol). The error bars correspond to the standard error of the mean computed over 20 different simulation runs.

For a single run, we thus have statistics for 17 values of *σ* that are regularly spaced between −0.08 and 0. The associated supercoiling rate, per sweep, of *σ* variation is given by v=-0.005/N, with $N=5\times 10^{5}$ and $N=1.6\times 10^{7}$ for the quickest and slowest simulations, respectively—for clarity, we normalize *v* such that *v* = 1 for the quickest simulations (Fig 5). Note that, in our simulations, a maximum of *M* = 100 cylinders can be rotated during a crankshaft rotation. Simulations being performed with a resolution of 30 bp per cylinder, a 30 kb long chain is made of *N* = 1000 cylinders such that a sweep corresponds to *N*/*M* = 10 Monte-Carlo steps. As a result, the slowest simulations with $N=1.6\times 10^{7}$ corresponds to $N_{MC}=1.6\times 10^{8}$ Monte-Carlo steps, so that one simulation run for the slowest case involves 17 × 1.6 × 10^8^ = 2.72 × 10^9^ Monte-Carlo steps.

For each simulation run, statistical analysis were performed by considering 2500 conformations between the (*N*/2)^*th*^ sweep (mid-total number of sweeps) and the *N*^*th*^ sweep (last sweep). In this regard, Fig 5 shows, for each supercoiling rate, the mean value of the radius of gyration as a function of *σ* together with the standard error of the mean. The latter is computed using the variance of the mean of the radii of gyration obtained from the 20 different simulation runs, i.e. $\sqrt{\text{var}(R_{g})/19}$ where var(*R*_*g*_) is that variance. Fig 5 shows in particular that for rates smaller than *v* = 1/8, results may be considered independent of *v*. As a consequence, quantities reported in this paper, such as *P*_*s*_(*r*), have been obtained using 60 independent simulation runs coming from the three slowest rates (*v* = 1/8, 1/16, 1/32) and considering 20 independent simulation runs.

### 6.2 Number of plectonemic branches

**Fig 6:**
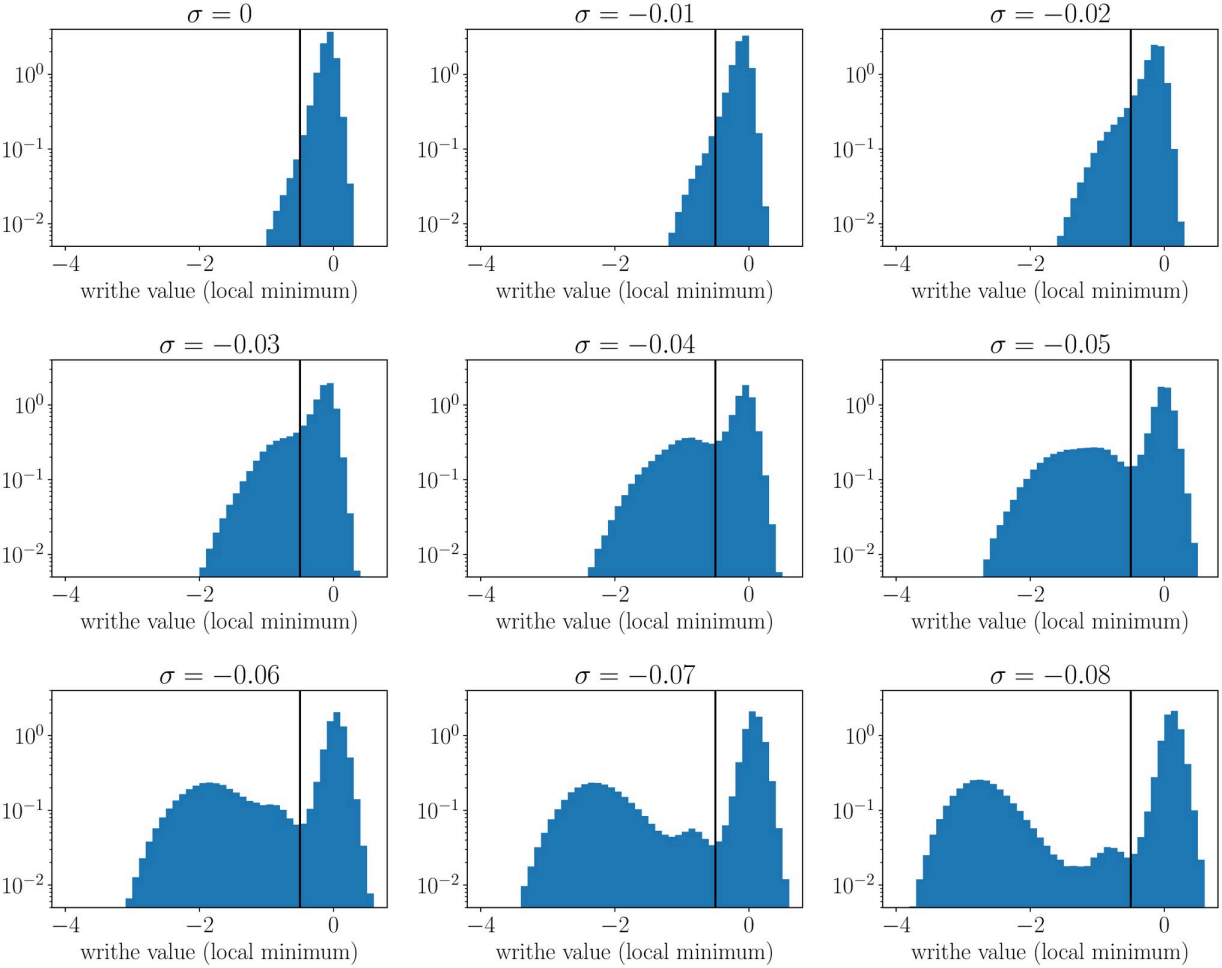
Distribution of local minima for the local writhe. Each plot was obtained for a given value of *σ*. The vertical lines indicate the threshold wr* below which local minima are considered to be associated with plectonemic branches.

The number of plectonemic branches (i.e. of external branches of the tree-like structures of supercoiled DNA) is computed using a local writhe, wr, as introduced in [22]. Specifically, for a given site *i* of the chain (*i* ∈ {1..*N*}), $\text{wr}\left(i\right)={\left(2\pi \right)}^{-1}\sum_{j=i-m/2}^{i+m/2}\Omega_{ij}$ where Ω_*ij*_ is given in Eqs. 16-21 of [25] (see [49] for the original derivation) and *m* defines the window over which the local writhe is computed. Here we take *m* = 10 such that it corresponds to two times the DNA bending persistence length—it is therefore expected to be sensitive to the smallest plectonemes (i.e. curls). Next, for each conformation, we compute its profile of local writhes, that is, we compute the curve (*i*, wr(*i*)) with *i* varying from 1 to *N*. We then identify the most significant local minima (largest negative values), which indicate *a priori* the presence of plectonemic branches. To this end, we compute distributions of all local minima over all studied conformations (Fig 6). From these distributions, we define a threshold, wr* (vertical black lines in Fig 6), that separates values associated with non-plectonemic DNA, on one hand, and values associated with plectonemes, on the other hand. Note that multimodal distributions are only present below *σ* ≃ −0.04, in accord with the observation that plectonemes do not manifest themselves for too small supercoiling values. Given wr*, the number of plectonemic branches of a conformation is given by the number of local minima with wr ≤ wr*.

### 6.3 The leaky cluster model

For the quenched cluster model, all ParB proteins are confined to a spherical volume with radius *ω*/2, what we refer to as a “cluster core” in the following. For the leaky cluster model, we consider an additional process where ParB proteins are produced at the edge of a cluster core and diffuse in the volume—the leaky core cluster radius is thus smaller than *ω*/2 in order to obtain the same full width at half maximum (*ω*) of *C*^(0)^ (see below). This production process accounts for the fact that, for the experimental conditions we consider, ParB proteins are produced in excess with respect to the capacity of the cluster core. The location of the source at the edge of the cluster may then model several situations (see main text for further details and references): either ParB production occurs close to the cluster as in the case of the plasmid or of membrane proteins that are often produced close to the membrane; or the cluster continuously “radiates” ParB proteins, which would be in accord with the prediction that ParB-ParB interactions are on the order of *k*_*B*_*T* inside the cluster. Since diffusing proteins are continuously diluted due to cell growth and cell division, we further consider an annihilation process. In this context, the *a priori* time-dependent quantity *C*^(0)^(*r*, *t*), which is itself proportional to the concentration of proteins, is governed by the following diffusion equation (in spherical coordinates):

$$\begin{array}{c} \partial_{t}C^{\left(0\right)}-\frac{D}{r^{2}}\partial_{r}[r^{2}\partial_{r}C^{\left(0\right)}]=S\left(r\right)-\Gamma \left(r\right)C^{\left(0\right)} \end{array}$$ (3)

where *S*(*r*) is the time-independent protein source contribution and Γ(*r*) is the time-independent dilution rate.

Here, we consider a source located at *r* = *ρ* (radius of the cluster core) and look for the solution when *r* > *ρ*, that is, where *S*(*r*) = 0. We also consider the simple case of a homogeneous dilution such that Γ(*r*) is independent of *r* (and equal to Γ). The corresponding stationary equation (where ∂_*t*_*C*^(0)^ = 0) reads:

$$\begin{array}{c} \frac{D}{r}[2\partial_{r}C^{\left(0\right)}+r\partial_{r}^{2}C^{\left(0\right)}]=\Gamma C^{\left(0\right)}. \end{array}$$ (4)

Writing *X*(*r*) = *rC*^(0)^(*r*), we have $D\partial_{r}^{2}X=\Gamma X$, whose solution reads *X*(*r*) = *A* exp [−*r*/*ξ*], with $\xi =\sqrt{\frac{D}{\Gamma }}$ and *A* a constant determined by the boundary conditions. As a consequence, the solution of Eq 4 reads:

$$\begin{array}{c} C^{\left(0\right)}\left(r\right)=A\frac{\exp[-r/\xi ]}{r}\;\text{with}\;\xi =\sqrt{\frac{D}{\Gamma }}, \end{array}$$ (5)

which is known as the Yukawa or screened Coulomb potential.

The question now concerns the value of *ξ*. To this end, we consider a lower bound for *D* equal to 1 *μm*^2^ s^−1^ and an upper bound for the dilution rate of 1000 proteins every ∼ 1000 s (one cell cycle in rapid growth). In this context, the lower bound for *ξ* is equal to ≃ 1000 nm, i.e. two times the radius of the cell. In practice, we thus consider *C*^(0)^(*r*) = *A*/*r* and specify the volume over which the integral is performed (see section 4.2 for details). To determine *A*, we consider that there is saturation at the edge of the cluster (*C*^(0)^(*ρ*) = 1) leading to *A* = *ρ*. Altogether, the leaky cluster model is thus defined by:

$$\begin{array}{ccc} C^{\left(0\right)}\left(r\right) & = & \left\{ \begin{array}{c} 1\;\text{if}\;r\leq \rho \\ \frac{\rho }{r}\;\text{if}\;r>\rho \end{array}\right. \end{array}$$ (6)

$$\begin{array}{ccc} \rho & = & \frac{\omega }{4} \end{array}$$ (7)

### 6.4 ChIP-Seq dataset

The ChIP-seq data were from a previous study [15] and are available on Gene Expression Omnibus GSE115274 (https://www.ncbi.nlm.nih.gov/geo/query/acc.cgi?acc=GSE115274). For the ParB density plots, the reads were counted at the center of each DNA fragment, relative to the average DNA fragment size determined for each library. The number of reads at any genomic position was then normalized after background subtraction and set to the value of 1 at the maximum intensity.

## Supporting information

S1 Text**Fig A. Below 10 kb, only slight differences exist between binding profiles obtained with 30 kb long molecules and those obtained with 60 kb long molecules**. **Left panel:** the blue, orange and green curves stand for the ratio of binding profiles between 30 kb and 60 kb long molecules obtained with different combinations of *σ*, *ω* and type of cluster. Red curve: ratio of binding profiles for a 30 kb long molecule with a leaky cluster and *ω* = 43 nm (best parameter for plasmid data) between *σ* = 0 and *σ* = −0.04. **Right panel:** We report the binding profiles used to compute the orange and red curves on the left panel to demonstrate that differences between 30 kb and 60 kb long molecules are indeed not significant from the viewpoint of experimental data (the purple curves are hardly distinguishable). By contrast, the difference is significative between *σ* = 0 (brown dashed curve) and *σ* = −0.04 (purple curves). **Fig B. Capturing chromosomal binding profiles**. Black curve: ChIP-seq chromosomal data. Smooth plain curves: best models using a quenched cluster (in orange) or a leaky cluster (in green). Smooth dashed curve: best model at *σ* = 0 with a leaky cluster. **Fig C. Testing Gaussian and exponential clusters.** We tested whether a Gaussian decay (top row) or an exponential decay (bottom row) could capture experimental profiles obtained on the chromosome (leftmost columns) or on the plasmid (rightmost columns). The heat maps correspond to Fig 2 of the main text, showing in particular that the best fit in both cases are found for a value of *ω* that is much larger that *ω*_*exp*_ (black horizontal bands). For the profiles, we compare the best match of the data in each case (orange curve) to the best match using the leaky cluster (black dashed curve). **Fig D. Snapshots of DNA conformation for various values of the supercoiling density (*σ*).** Below *σ* = −0.04 (bottom row), one can observe well-defined plectonemes, which become tighter and longer as *σ* further decreases. The green spheres are used to indicate the length scales associated with the decrease of $C_{L}^{\left(0\right)}\left(r\right)$ for the leaky cluster case. Namely, the three spheres respectively have a diameter equal to 40 nm (close to the value of *ω*_*exp*_), 80 nm and 160 nm, which correspond to $C_{L}^{\left(0\right)}$ equal to ≃1/2, 1/4, 1/8. Note that *parS* (the center of the spheres) has been placed here in the interior of the fold to better indicate the lengths at play. However, in our model, it can be located anywhere along the DNA (e.g. at the apex of a plectoneme) as no experimental information is available to constrain the model. Note: the visualization of the conformations was based on Mathematica^®^ using the Tube function with a spline effect (SplineDegree → 3). **Fig E. The different cases to consider to compute *C*(*r*) as a function of *C*^(*o*)^(*x*).** *P* indicates a point at distance *r* from *parS* at which we compute *C*(*r*). The small red circle and arcs of a circle indicate possible locations of the center of the cluster knowing it is located at a distance *x* from *P* (and, hence, contributing by *C*^(*o*)^(*x*)). The large dashed red circle indicates the maximal distance between *parS* and the center of the cluster core. A) The distance *r* and *x* are such that all the positions on the *P*-centered sphere of radius *x* are possible for the center of the cluster core, leading to $\Pi_{r}\left(x\right)=\Pi_{r}^{\left(1\right)}\left(x\right)=3x^{2}/\rho^{3}$. B) *P* is located inside the volume accessible by the cluster core but *x* is large enough such that only part of the *P*-centered sphere of radius *x* contributes to the signal, leading to $\Pi_{r}\left(x\right)=\Pi_{r}^{\left(2\right)}\left(x\right)=\frac{3x}{4r\rho^{3}}(\rho^{2}-{\left(r-x\right)}^{2})$. C) *P* is located outside the volume accessible by the cluster core such that, just as in B, $\Pi_{r}\left(x\right)=\Pi_{r}^{\left(2\right)}\left(x\right)$.(PDF)
