# Supplementary material for: Supercoiled DNA and non-equilibrium formation of protein complexes: A quantitative model of the nucleoprotein ParBS partition complex
Source: PLoS Comput Biol. 2021 Apr 16;17(4):e1008869. doi: 10.1371/journal.pcbi.1008869 (PMC8092679; doi:10.1371/journal.pcbi.1008869)
Supplement: S1 Text — Fig A. Below 10 kb, only slight differences exist between binding profiles obtained with 30 kb long molecules and those obtained with 60 kb long molecules. Left panel: the blue, orange and green curves stand for the ratio of binding profiles between 30 kb and 60 kb long molecules obtained with different combinations of σ, ω and type of cluster. Red curve: ratio of binding profiles for a 30 kb long molecule with a leaky cluster and ω = 43 nm (best parameter for plasmid data) between σ = 0 and σ = −0.04. Right panel: We report the binding profiles used to compute the orange and red curves on the left panel to demonstrate that differences between 30 kb and 60 kb long molecules are indeed not significant from the viewpoint of experimental data (the purple curves are hardly distinguishable). By contrast, the difference is significative between σ = 0 (brown dashed curve) and σ = −0.04 (purple curves). Fig B. Capturing chromosomal binding profiles. Black curve: ChIP-seq chromosomal data. Smooth plain curves: best models using a quenched cluster (in orange) or a leaky cluster (in green). Smooth dashed curve: best model at σ = 0 with a leaky cluster. Fig C. Testing Gaussian and exponential clusters. We tested whether a Gaussian decay (top row) or an exponential decay (bottom row) could capture experimental profiles obtained on the chromosome (leftmost columns) or on the plasmid (rightmost columns). The heat maps correspond to Fig 2 of the main text, showing in particular that the best fit in both cases are found for a value of ω that is much larger that ωexp (black horizontal bands). For the profiles, we compare the best match of the data in each case (orange curve) to the best match using the leaky cluster (black dashed curve). Fig D. Snapshots of DNA conformation for various values of the supercoiling density (σ). Below σ = −0.04 (bottom row), one can observe well-defined plectonemes, which become tighter and longer as σ further decreases. The green spheres are used to ind [file pcbi.1008869.s001.pdf]

Supercoiled DNA and non-equilibrium formation of protein complexes: a quantitative model of the nucleoprotein ParBS partition complex

Jean-Charles Walter[1,*], Thibaut Lepage[2], Jérôme Dorignac[1], Frédéric Geniet[1], Andrea Parmeggiani[1,3], John Palmeri[1], Jean-Yves Bouet[4] and Ivan Junier[2,*]
[1]*Laboratoire Charles Coulomb (L2C), Univ. Montpellier, CNRS, Montpellier, France*
[2]*CNRS, Univ. Grenoble Alpes, TIMC-IMAG, Grenoble, France*
[3]*LPHI, Univ. Montpellier, CNRS, Montpellier, France and*
[4]*LMGM, CBI, CNRS, Univ. Toulouse, UPS, Toulouse, France*[*]

CONTENTS

I. COMPUTING $C(r)$ FROM THE KNOWLEDGE OF $C^{(0)}(x)$

Here, we aim at computing the probability $C(r)$ to find a ParB protein at a point P located at a distance r of *parS*, knowing that the center of the cluster can be found anywhere in the *parS*-centered sphere with radius ρ (dashed red circle in Fig E, with $\rho = \omega/2$ and $\rho = \omega/4$ for the cases of the quenched and leaky clusters, respectively (see above and main text for the definition of ω). Specifically, given the probability $\Pi_r(x)$ to find the cluster center at a distance x of P, knowing that the latter is located at distance r of *parS*, and given the probability $C^{(0)}(x)$ to find a ParB protein at a distance x of the cluster center, $C(r)$ can be written as:

$$C(r) = \int_0^{\infty} dx \Pi_r(x) C^{(0)}(x), \tag{1}$$

We next consider the general situation where $C^{(0)}(r) = \theta(\rho - r) + \theta(r - \rho) \times f(r)$ where: i) $\rho = \omega/2$ and $f(r) = 0$ for the quenched cluster and ii) $\rho = \omega/4$ and $f(r) = \rho/r$ for the leaky cluster. Considering separately the cases $r \leq \rho$ and $r \geq \rho$, one can then show that $C(r)$ reads:

$$C(r) = \theta(\rho - r)\left[\int_0^{\rho-r} dx \Pi_r^{(1)}(x) C^{(0)}(x) + \int_{\rho-r}^{\rho+r} dx \Pi_r^{(2)}(x) C^{(0)}(x)\right] + \theta(r - \rho)\int_{\rho-r}^{\rho+r} \Pi_r^{(2)}(x) C^{(0)}(x) \tag{2}$$

$\Pi_r^{(1)}(x) = 3x^2/\rho^3$ (which is independent of r) stands for the probability density to pick a point on the P-centered sphere with radius x given a random process of picking points uniformly in the *parS*-centered spherical volume with radius ρ, knowing that the former is entirely included in the latter, i.e. knowing that $r + x \leq \rho$ (Fig E(A)). $\Pi_r^{(2)}(x) = \frac{3x}{4r\rho^3}\left(\rho^2 - (r - x)^2\right)$ is the corresponding probability in the situation where either $r \leq \rho$ and $r + x \geq \rho$ (Fig E(B)) or $r \geq \rho$ and $r - \rho \leq x \leq r + \rho$ (Fig E(C)), i.e. when the P-centered sphere intersects only partially with the *parS*-centered spherical volume.

Interestingly, one can check that considering $C^{(0)}(r)$ as an approximation of $C(r)$ leads to similar results, with significant differences only for small binding probabilities. As a consequence, results presented in Figs 2 and 3 of the main text are qualitatively similar when using $C^{(0)}(r)$ in place of $C(r)$, with in particular the same values of best parameters.

* jean-charles.walter@umontpellier.fr; ivan.junier@univ-grenoble-alpes.fr

A. Quenched cluster: $C_Q^{(0)}(x) = \theta(\rho - x)$

In this case, using the notation $W_{r_1}^{r_2}(r) = \theta(r_2 - r) \times \theta(r - r_1)$ for the window function, we find:

$$C_Q(r) = W_0^\rho(r)\left[\left(\frac{\rho - r}{r}\right)^3 + P_r^{(2)}(\rho) - P_r^{(2)}(\rho - r)\right] + W_\rho^{2\rho}(r)\left[P_r^{(2)}(\rho) - P_r^{(2)}(r - \rho)\right] \tag{3}$$

$$P_r^{(2)}(x) = \int^x \Pi_r^{(2)}(y)dy = \frac{3}{8}\frac{x^2}{r^2}\left(\frac{r}{\rho} - \left(\frac{r}{\rho}\right)^3\right) + \frac{1}{2}\frac{x^3}{\rho^3} - \frac{3}{16}\frac{x^4}{r\rho^3} \tag{4}$$

$$\rho = \omega/2 \tag{5}$$

B. Leaky cluster: $C_L^{(0)}(x) = \theta(\rho - x) + \theta(x - \rho) \times \rho/x$

In this case, we find:

$$\begin{aligned} C_L(r) = & W_0^\rho(r)\left[\left(\frac{\rho - r}{r}\right)^3 + P_r^{(2)}(\rho) - P_r^{(2)}(\rho - r) + Q_r^{(2)}(r + \rho) - Q_r^{(2)}(\rho)\right] + \\ & W_\rho^{2\rho}(r)\left[P_r^{(2)}(\rho) - P_r^{(2)}(r - \rho) + Q_r^{(2)}(r + \rho) - Q_r^{(2)}(\rho)\right] + \\ & W_{2\rho}^{\infty}(r)\left[Q_r^{(2)}(r + \rho) - Q_r^{(2)}(r - \rho)\right] \end{aligned} \tag{6}$$

$$P_r^{(2)}(x) = \int^x \Pi_r^{(2)}(y)dy = \frac{3}{8}\frac{x^2}{r^2}\left(\frac{r}{\rho} - \left(\frac{r}{\rho}\right)^3\right) + \frac{1}{2}\frac{x^3}{\rho^3} - \frac{3}{16}\frac{x^4}{r\rho^3} \tag{7}$$

$$Q_r^{(2)}(x) = \int^x \frac{\rho}{y}\Pi_r^{(2)}(y)dy = \frac{3}{4}\frac{x}{r}\left(1 - \left(\frac{r}{\rho}\right)^2\right) + \frac{3}{4}\frac{x^2}{\rho^2} - \frac{1}{4}\frac{x^3}{r\rho^2} \tag{8}$$

$$\rho = \omega/4 \tag{9}$$

II. SUPPLEMENTARY FIGURES

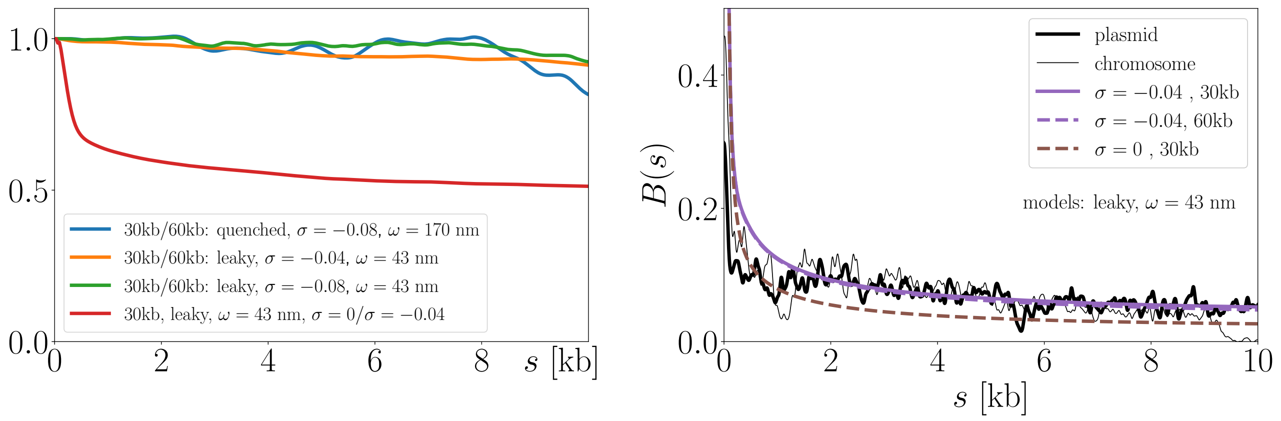

Fig A: *Below* 10 kb*, only slight differences exist between binding profiles obtained with* 30 kb *long molecules and those obtained with* 60 kb *long molecules.* **Left panel:** the blue, orange and green curves stand for the ratio of binding profiles between 30 kb and 60 kb long molecules obtained with different combinations of σ, ω and type of cluster. Red curve: ratio of binding profiles for a 30 kb long molecule with a leaky cluster and $\omega = 43$ nm (best parameter for plasmid data) between $\sigma = 0$ and $\sigma = -0.04$. **Right panel:** We report the binding profiles used to compute the orange and red curves on the left panel to demonstrate that differences between 30 kb and 60 kb long molecules are indeed not significant from the viewpoint of experimental data (the purple curves are hardly distinguishable). By contrast, the difference is significative between $\sigma = 0$ (brown dashed curve) and $\sigma = -0.04$ (purple curves).

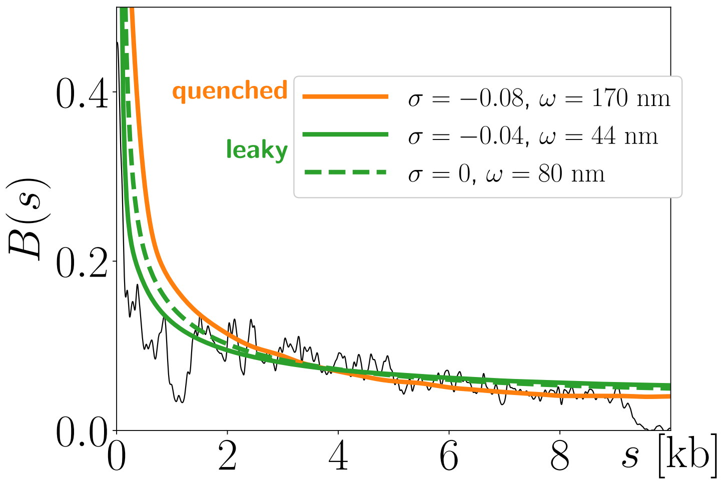

Fig B: *Capturing chromosomal binding profiles.* Black curve: ChIP-seq chromosomal data. Smooth plain curves: best models using a quenched cluster (in orange) or a leaky cluster (in green). Smooth dashed curve: best model at $\sigma = 0$ with a leaky cluster.

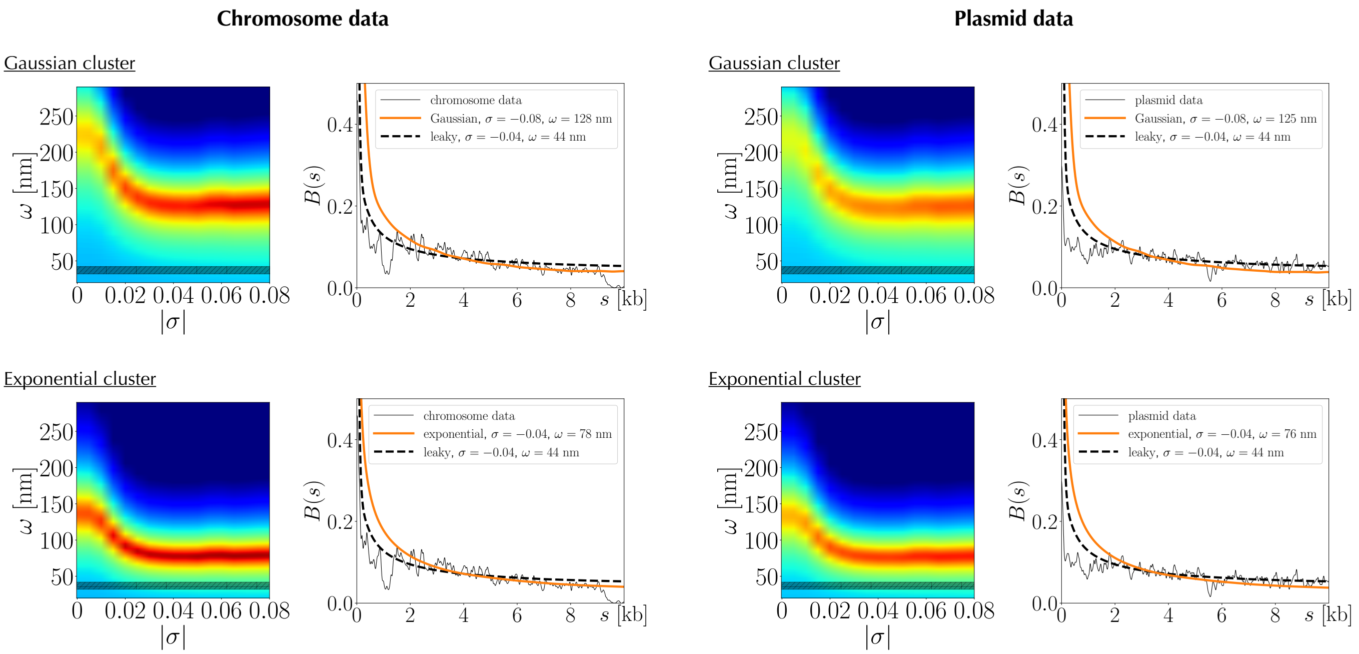

Fig C: *Testing Gaussian and exponential clusters.* We tested whether a Gaussian decay (top row) or an exponential decay (bottom row) could capture experimental profiles obtained on the chromosome (leftmost columns) or on the plasmid (rightmost columns). The heat maps correspond to Fig 2 of the main text, showing in particular that the best fit in both cases are found for a value of ω that is much larger that ω_{exp} (black horizontal bands). For the profiles, we compare the best match of the data in each case (orange curve) to the best match using the leaky cluster (black dashed curve).

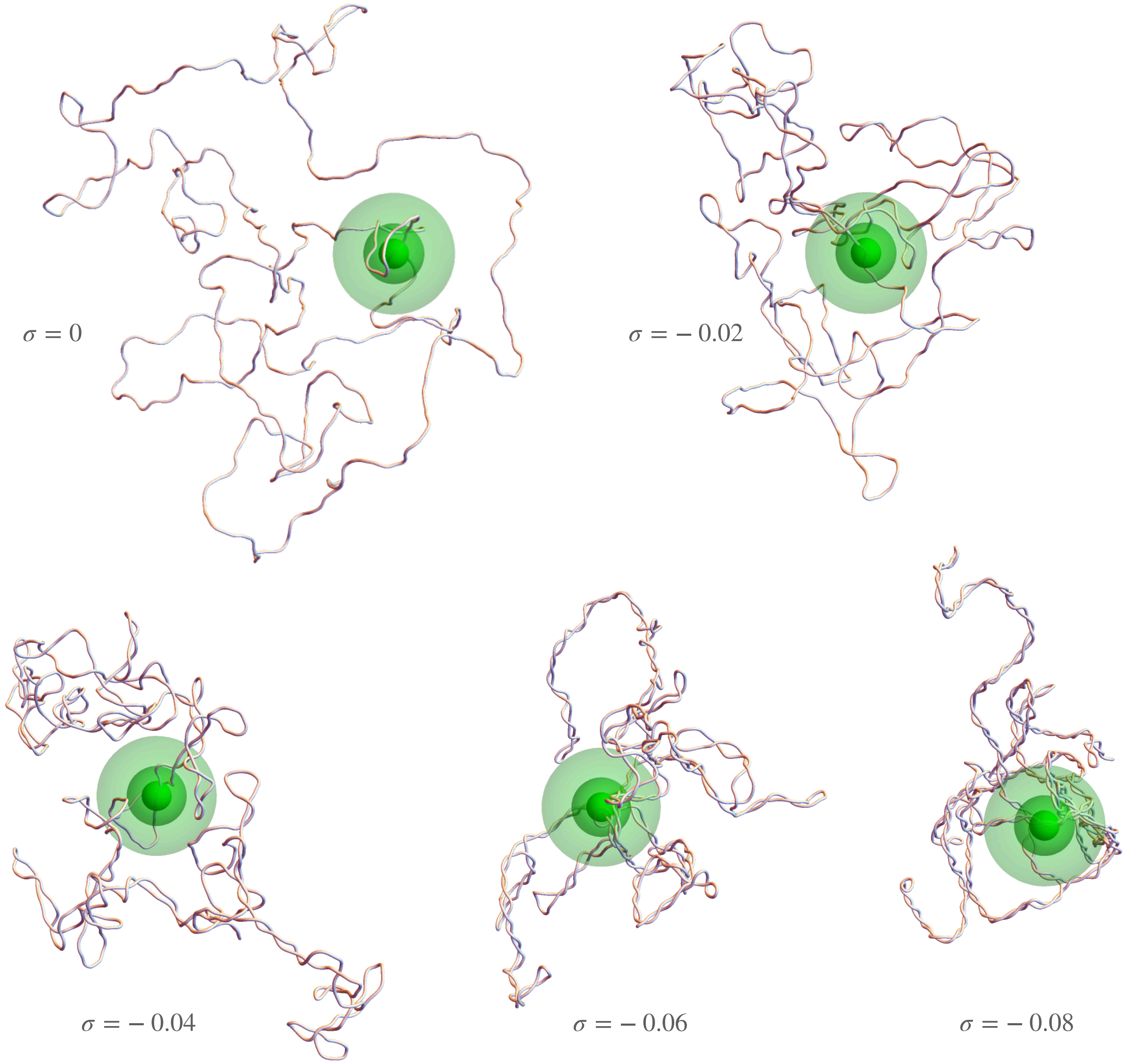

Fig D: *Snapshots of DNA conformation for various values of the supercoiling density (σ).* Below $\sigma = -0.04$ (bottom row), one can observe well-defined plectonemes, which become tighter and longer as σ further decreases. The green spheres are used to indicate the length scales associated with the decrease of $C_L^{(0)}(r)$ for the leaky cluster case. Namely, the three spheres respectively have a diameter equal to 40 nm (close to the value of ω_{exp}), 80 nm and 160 nm, which correspond to $C_L^{(0)}$ equal to $\simeq 1/2, 1/4, 1/8$. Note that *parS* (the center of the spheres) has been placed here in the interior of the fold to better indicate the lengths at play. However, in our model, it can be located anywhere along the DNA (e.g. at the apex of a plectoneme) as no experimental information is available to constrain the model. Note: the visualization of the conformations was based on Mathematica® using the Tube function with a spline effect (SplineDegree → 3).

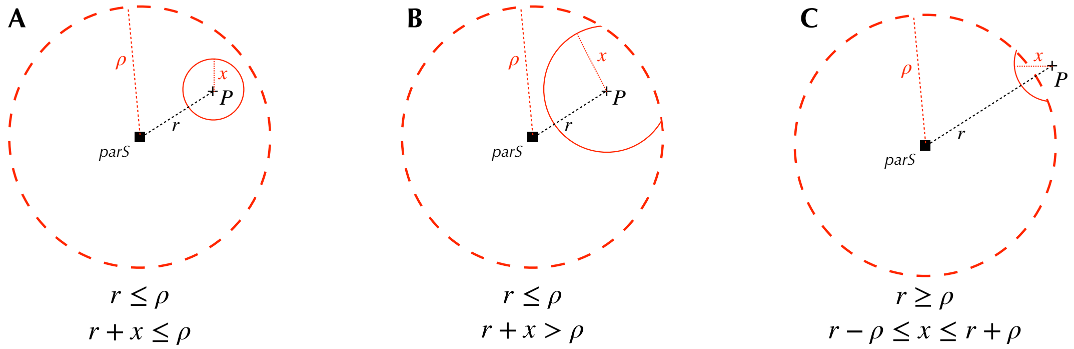

Fig E: *The different cases to consider to compute $C(r)$ as a function of $C^{(o)}(x)$.* P indicates a point at distance r from *parS* at which we compute $C(r)$. The small red circle and arcs of a circle indicate possible locations of the center of the cluster knowing it is located at a distance x from P (and, hence, contributing by $C^{(o)}(x)$). The large dashed red circle indicates the maximal distance between *parS* and the center of the cluster core. A) The distance r and x are such that all the positions on the P-centered sphere of radius x are possible for the center of the cluster core, leading to $\Pi_r(x) = \Pi_r^{(1)}(x) = 3x^2/\rho^3$. B) P is located inside the volume accessible by the cluster core but x is large enough such that only part of the P-centered sphere of radius x contributes to the signal, leading to $\Pi_r(x) = \Pi_r^{(2)}(x) = \frac{3x}{4r\rho^3}\left(\rho^2 - (r-x)^2\right)$. C) P is located outside the volume accessible by the cluster core such that, just as in B, $\Pi_r(x) = \Pi_r^{(2)}(x)$.
